# STK35 Is Ubiquitinated by NEDD4L and Promotes Glycolysis and Inhibits Apoptosis Through Regulating the AKT Signaling Pathway, Influencing Chemoresistance of Colorectal Cancer

**DOI:** 10.3389/fcell.2020.582695

**Published:** 2020-10-08

**Authors:** Haojun Yang, Jie Zhu, Guangyao Wang, Hanyang Liu, Yan Zhou, Jun Qian

**Affiliations:** Department of Gastrointestinal Center, The Affiliated Changzhou No. 2 People’s Hospital of Nanjing Medical University, Changzhou, China

**Keywords:** STK35, ubiquitination, colorectal cancer, AKT, apoptosis

## Abstract

The development of colorectal cancer (CRC) is often sporadic, but its etiology is multifactorial. Chemoresistance of CRC leads to tumor recurrence and poor prognosis in patients. The phosphorylation of protein kinase B (AKT) can activate metabolic reprogramming toward cellular glycolysis. Serine/threonine kinase 35 (STK35) regulates the cell cycle and is frequently associated with cancer progression, whereas little is known about its specific roles in CRC. In the current study, bioinformatics analyses were performed to investigate the relationship between STK35 and CRC prognosis. STK35 knockdown and overexpressing CRC cells were established to examine its functions in CRC. Fluorouracil (5-FU) was utilized to evaluate the effect of STK35 on CRC chemoresistance. Moreover, co-immunoprecipitation was performed to explore the ubiquitination of STK35. STK35 was highly expressed in CRC, and its protein expression was negatively correlated with the survival of CRC patients. Furthermore, STK35 overexpression could promote glycolysis, suppress apoptosis, upregulate p-AKT, and counteract the antitumor functions of 5-FU and neural precursor cell expressed developmentally downregulated gene 4-like (NEDD4L) in CRC cells. NEDD4L was associated with and could ubiquitinate STK35. STK35 could be a prognostic biomarker for CRC prognosis and has promotive effects on CRC cellular activities, partially through the AKT pathway. Moreover, STK35 also interferes with the chemosensitivity of CRC.

## Introduction

Colorectal cancer (CRC) remains as one of the most common malignancies, as well as the primary cause of cancer-related deaths worldwide, and its incidence is increasing rapidly among teenagers and adults (Arnold et al., 2017; Siegel et al., 2017). Multiple risk factors, including hereditary components, lifestyle patterns, dietary styles, and environmental influences, are capable of inducing the development and progression of CRC (Brenner et al., 2014). While the tumorigenesis of CRC is commonly in stepwise mode as sporadic (Fearon and Vogelstein, 1990). Until now, therapeutic approaches, especially chemotherapies, for CRC have been established by targeting the suppression of cell apoptosis and advanced metabolism. However, the intrinsic or acquired chemoresistance of CRC malignant cells restricts the efficacy of chemo-reagents, leading to tumor recurrence and further metastasis (Zheng, 2017). The poor treatment outcomes and prognosis for CRC contribute to the relatively low survival probability of CRC patients, and the current mortality rate ranges from 12 to 87% (stage IV to I) (Cronin et al., 2018).

Generally, CRC cells possess a unique phenotype of global metabolic reprogramming, undergoing aerobic glycolysis (known as the “Warburg effect”), for which glucose serves as the dominant energy source; tumor cells can utilize glucose for the production of pyruvate and adenosine triphosphates (Brown et al., 2018). During the glycolytic process, numerous metabolic intermediates or by-products support the synthesis of macromolecules for rapid cell proliferation (Vander Heiden et al., 2009). Moreover, certain intracellular signaling pathways of glycolysis with genetic drivers also influence other features of cancerous cells (La Vecchia and Sebastian, 2020); for example, the hyperactive phosphatidylinositol-3-OH kinase (PI3K) signal stimulates cell growth (Yu and Grady, 2012), while aberrant tumor protein p53 signaling inhibits apoptosis (Slattery et al., 2019).

The PI3K/protein kinase B (AKT) signaling transduction cascade mediated cell-cycle regulation, as well as proliferation, apoptosis, and differentiation (Osaki et al., 2004). During stable energy conditions, the cellular PI3K/AKT pathway remains catalytically inactive or with limited activity, which ensures a quiescent state for the maintenance of normal cell cycle (Cheung and Rando, 2013; Hung et al., 2017). However, when the cellular glucose level increases, induced growth factors, such as epidermal growth factor, insulin, and insulin-like growth factor, can further activate PI3K, which then phosphorylates and activates AKT (Danielsen et al., 2015). The activation of the PI3K/AKT signaling pathway is associated with nearly 70% of CRC cases, while at the same time, inhibition of the signaling pathway is considered a target for CRC therapy (Malinowsky et al., 2014). In fact, the activated p-AKT can sequentially phosphorylate multiple regulatory proteins, such as cytosolic B cell lymphoma 2-associated death promoter, glycogen synthase kinase 3β, and mouse double minute 2 homolog, as well as nuclear forehead box protein O (Manning and Cantley, 2007). All of these p-AKT downstream proteins are key regulators of normal cell cycle and carcinogenesis, particularly the control of apoptosis and energy metabolism (Nitulescu et al., 2018). Therefore, p-AKT serves as the core factor in the PI3K/AKT signaling pathway, which indicates oncogenic transformation in cells (Chang et al., 2003).

Serine/threonine kinases (STKs) play critical roles through the phosphorylation and activation of relevant effectors, such as cell-cycle regulators, growth factors, and transcription activators, to regulate signaling pathways and cellular homeostasis (Manning et al., 2002; Capra et al., 2006). However, dysregulation of STKs can lead to a deregulated cell cycle, in favor of unlimited cell proliferation, repressed cell apoptosis, and minimized cell differentiation, which facilitate the development of tumors and subsequent metastasis (Freeman and Whartenby, 2004). Although the detailed biological functions of STK35 are still being investigated, STK35 has regulatory roles in cell-cycle modulation, and its abnormal cellular levels are implicated in various human diseases including cancer (Vallenius and Makela, 2002; Goyal et al., 2009). It has also been reported that STK35 is associated with programmed cell death (Yasuda et al., 2012), and *STK35* gene expression is altered in Parkinson disease (Hourani et al., 2008). Furthermore, STK35 is essential for the angiogenesis and migration of endothelial cells (Goyal et al., 2011), as well as cellular apoptosis and proliferation of osteosarcoma (Wu et al., 2018). Moreover, the upregulation of *STK35* has been suggested to be linked with human CRC (Capra et al., 2006), whereas the explicit relationship between them and the specific functions of STK35 in CRC have not been systematically studied.

In the current study, we proposed to explore the correlation between STK35 and prognostic conditions in CRC patients, the roles of STK35 in CRC cellular activities and tumor development, and the possible mechanisms underlying the functions of STK35. We demonstrate that STK35 is highly expressed in CRC tumor tissues and that its expression is positively correlated with the mortality rate of CRC patients. Furthermore, through establishing STK35 knockdown and overexpression in CRC cells, we also reveal that STK35 can interfere with the chemo-sensitivity of CRC cells. We also demonstrate that STK35 promotes both *in vitro* cellular activities and *in vivo* tumor growth of CRC, potentially through regulating the AKT signaling pathway. In addition, TK35 is ubiquitinated by neural precursor cell expressed developmentally downregulated gene 4-like (NEDD4L) and can also counteract the anti-CRC effects of NEDD4L.

## Materials and Methods

### Bioinformatics Analysis

RNA-seq data related to the expression of STK35 and NEDD4L in various cancer patients were acquired from The Cancer Genome Atlas (TCGA) database, including 638 cases for colorectal tumor tissues and 51 cases for non-tumor tissues in patients with CRC, and the GEO database (access id: GSE9348), including 70 cases of tumor tissues and 12 cases of normal tissues. The gene set enrichment analysis (GSEA) algorithm was used to identify pathways that were significantly enriched between STK35 high vs. low expression.

### Clinical Samples

A total of 131 CRC patients in The Affiliated Changzhou No. 2 People’s Hospital of Nanjing Medical University were enrolled between March 2013 and October 2015. Tumor tissues and their corresponding non-cancerous tissues were collected for storage at −80°C until further analysis. The study was approved by the medical ethics committee of The Affiliated Changzhou No. 2 People’s Hospital of Nanjing Medical University, and the study was conducted according to the Declaration of Helsinki. All patients provided written informed consent prior to participation.

### Quantitative Real Time PCR (Q-PCR)

Total RNA was extracted from cells or tissues by TRIzol reagent (Life Technologies, United States) and reverse transcribed to cDNA with PrimeScript kit (Takara Biotechnology, China) in accordance with the manufacturers’ protocols. Quantitative real time PCR (Q-PCR) was carried out using SYBR Green PCR Master Mix (Applied Biosystems, United States) on an ABI 9700 real-time PCR system (Applied Biosystems, United States). The primers used were as follows: STK35-F: 5′-CCTGAAGCCAGACAACATCC-3′, STK35-R: 5′-GT CTTGATTGCCCTCTTTGC-3′; NEDD4L-F: 5′-CTCGGTGAT GTGGATGTG-3′, NEDD4L-R: 5′-TTCGGCGTCCATGAGTA G-3′; and β-actin-F: 5′-TGGCATCCACGAAACTAC-3′, β-actin-R: 5′-CTTGATCTTCATGGTGCTG-3′. The fold changes at the transcript level were GAPDH-normalized and calculated based on the 2^–ΔΔCT^ method.

### Immunohistochemistry (IHC)

Formalin-fixed and paraffin-embedded CRC specimens were used for Immunohistochemistry (IHC) staining, as previously described (Zhu et al., 2017). In brief, the target tissues were deparaffinized and rehydrated, followed by heat-induced antigen retrieval with pH 8.0 EDTA. The slides were then stained with primary antibodies (Abcam, United States), including ab237517 against STK35, ab46521 against NEDD4L, and ab81283 against p-AKT, followed by incubation with horseradish peroxidase (HRP)-conjugated anti-IgG secondary antibody D-3004 (Long Island Biotech, China). Immunoreactivity was scored by two investigators using the H-score system based on the percentage of positively stained tumor cells. All patients with more than 25% of positively stained tumor cells were grouped as high-expression, while those with less than 25% were grouped as low-expression.

### Cell Culture

Human-origin CRC cell lines (HCT116, LOVO, SW480, SW620, and SW1116) and the normal human intestinal crypt cell line HIEC were obtained from the cell bank of Shanghai Biology Institute (Chinese Academy of Sciences) and cultured in a 5% CO_2_ incubator at 37°C. LOVO, SW480, SW620, and SW1116 cells were cultured in RPMI-1640 medium (Life Technologies, United States) supplemented with 10% fetal bovine serum (FBS) (Life Technologies, United States) and 1% penicillin/streptomycin. HCT116 cells were maintained in Dulbecco’s Modified Eagle Medium (Life Technologies, United States) supplemented as above.

### Gene Overexpression and Knockdown

Serine/threonine kinase 35 and NEDD4L overexpression plasmids were constructed by cloning the coding sequence of STK35 or NEDD4L into pLVX-Puro vectors (Takara Bio Inc., United States). To generate the knockdown clones, synthesized shRNA oligos targeting STK35 were cloned in pLKO.1 plasmids (Addgene, United States). Recombinant plasmids, together with the packaging/envelope plasmids psPAX2 and pMD2.G, were co-transfected to human CRC cells with Lipofectamine 2000 (Invitrogen, United States) in accordance with the manufacturer’s instructions. The virus particles were collected after 48 h post-transfection and further transfected into the cells of interest to generate overexpression and knockdown cell lines. The cells transfected with scramble shRNA (shNC) or blank plasmid (Vector) were considered as negative controls.

### Western Blot

Proteins were extracted with RIPA lysis buffer with mixed protease inhibitors (Sigma, United States). Proteins (30 μg) were separated on SDS-PAGE gel, followed by transfer to nitrocellulose membrane (MilliporeSigma, United States). The membranes were then blocked with 5% skim milk and incubated at 4°C overnight with the primary antibody: anti-STK35 (ab136695; Abcam, United States), anti-NEDD4L (#2740; Cell Signaling Technology, United States), anti-cleaved caspase-3 (ab2302; Abcam, Cambridge, MA, United States), anti-cleaved caspase-9 (ab2324; Abcam, United States), anti-GLUT1 (ab40084; Abcam, United States), anti-HK-2 (ab209847; Abcam, United States), anti-AKT (#9272; Cell Signaling Technology, United States), anti-p-AKT (#4060; Cell Signaling Technology, United States), or β-actin (#3700; Cell Signaling Technology, United States). Following primary antibody incubation, the membranes were incubated with HRP-conjugated secondary antibody (Beyotime, China). The membranes were visualized by using ChemiDoc Imaging Systems (Bio-Rad, United States).

### Cell Viability Assay

Cell viability was analyzed with Cell Counting Kit-8 (CCK-8) (SAB, United States) according to the manufacturer’s instructions. Briefly, HCT116, SW480, and SW620 cells transduced with the indicated plasmids were plated in 96-well plates (approximately 3,000 cells/well) and incubated overnight at 37°C, followed by CCK-8 incubation for 1 h at 37°C. The 450-nm optical density (OD) was determined using a multi-plate reader (DNM-9602; Perlong Medical Co., China).

### Cell Apoptosis Assay

HCT116, SW480, and SW620 cells were grown in 6-well plates (approximately 5 × 10^5^ cells/well) until they reached 50% confluence. Then, the cells expressing the indicated plasmids were treated with or without 10 μM of AKT inhibitor MK-2206, 200 or 400 μM of fluorouracil (5-FU), or vehicle control for 48 h. Following incubation with 5 μl propidium iodide (PI) and fluorescein isothiocyanate-labeled annexin V (Annexin V-FITC), cell apoptosis was assessed with a FACSArial I flow cytometer (BD Biosciences, United States). Cells that were negative for PI but positive for Annexin V-FITC were counted as apoptotic.

### Cellular Activity of Lactate Dehydrogenase (LDH)

The lactate dehydrogenase (LDH) activity in the cell-free cultural supernatants of HCT116, SW480, and SW620 cells was evaluated using a commercial assay kit A020-1 (Nanjing Jiancheng Bioengineering Institute, China) based on the protocol provided by the manufacturer.

### Extracellular Flux (XF) Analysis

The XF24 Extracellular Flux Analyzer (Seahorse, United States) was used to monitor extracellular acidification rates (ECAR) and cellular oxygen consumption rates (OCR) in real time for the estimations of glycolysis and mitochondrial respiration, respectively, as previously described (Guo et al., 2020).

### Establishment of Stable Cell Lines for Xenograft Study

Approximately 5 × 10^6^ SW620 cells transduced with shSTK35 or shNC were subcutaneously injected into the armpits of 4- to 5-week-old male nude mice (Beijing HFK Bioscience, China). Tumor measurements were recorded every 3 days. At the 33rd day post-injection, the mice were euthanized, their tumor characteristics were recorded, and the xenografts were collected for further analysis (*n* = 6 per group). The xenografts were subjected to terminal deoxynucleotidyl transferase dUTP nick end labeling (TUNEL). Meanwhile, approximately 5 × 10^6^ SW480 cells transduced with STK35-overexpressing lentivirus or blank lentivirus vector were injected into the same type of mice following the same method. Chemotherapy by 50 mg/kg of 5-FU (once per week) was initiated 12 days after the injection (*n* = 6 per group). At the 33rd day post-injection, the mice were sacrificed for measurement of the tumor size. A total of 80 mice were collected for survival analysis (*n* = 20 per group). Laboratory animal care and procedures were conducted following the animal ethics guidelines of The Affiliated Changzhou No. 2 People’s Hospital of Nanjing Medical University.

### Immunoprecipitation (IP) and Liquid Chromatography/Mass Spectrometry (LC/MS) Analyses

Cells stably expressing the empty vector or FLAG-tagged STK35 were lysed in precooled RIPA lysis buffer. The cell lysates were then incubated with protein A/G beads (Santa Cruz Biotechnology) for 2 h at 4°C, and the extracts were further incubated overnight with anti-FLAG beads (Sigma-Aldrich) at 4°C. Subsequently, the immunoprecipitated protein complex was eluted using the FLAG peptide (Sigma-Aldrich). Protein samples were resolved by SDS-PAGE for Coomassie Blue staining, and the differentially migrated bands were excised and digested with trypsin for further liquid chromatography/mass spectrometry (LC/MS) analysis.

### Co-immunoprecipitation (Co-IP) and Ubiquitination Assays

Cell lysates extracted with RIPA buffer were incubated with anti-STK35 antibody PA5-14082 (Invitrogen), anti-NEDD4L antibody #4013 (Cell Signaling Technology, United States), or IgG antibody sc-2027 (Santa Cruz Biotechnology, United States) overnight at 4°C, followed by 2 h incubation with Protein A/G PLUS-Agarose beads sc-2003 (Santa Cruz Biotechnology, Inc.) at 4°C. The immune-complexes were washed three times with lysis buffer on a magnetic rack and then examined by immunoblotting with anti-STK35 (ab136695; Abcam, United States), anti-NEDD4L (#2740; Cell Signaling Technology, United States), or anti-ubiquitin (ab7780; Abcam, Cambridge, MA, United States) antibodies.

### Statistical Analysis

All assays were performed three times, and the quantitative data are displayed as mean ± standard deviation. Statistical analysis was performed by GraphPad Prism 7.0 (GraphPad Software, United States). The comparison between different experimental groups was performed with unpaired *t*-test or ANOVA. Cox’s proportional hazards regression model and Kaplan–Meier plotting were applied for calculating the overall or disease-free survival, and the differences between groups were analyzed by a log-rank test. *P*-values < 0.05 were considered statistically significant.

## Results

### STK35 Expression Was Clinically Correlated With CRC Prognosis

**FIGURE 1:**
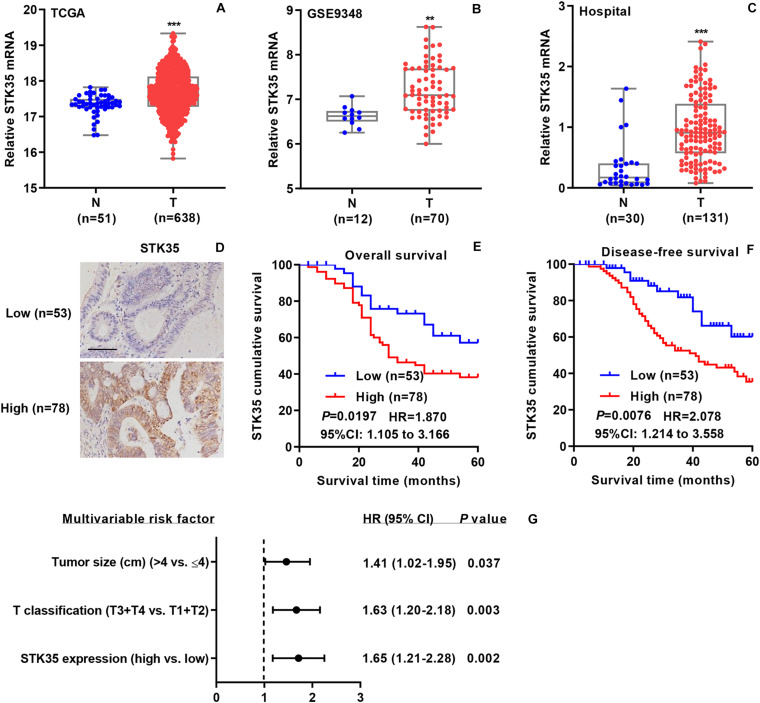
STK35 is clinically relevant in colorectal cancer (CRC). **(A)** STK35 mRNA expression in 638 CRC tissues (T) and 51 normal tissues (N) acquired from TCGA RNA-seq datasets. **(B)** STK35 mRNA expression in 70 CRC tissues (T) and 12 normal tissues (N) acquired from the GSE9348 database. **(C)** STK35 mRNA expression by quantitative RT-PCR in 131 CRC tissues (T) and 30 normal tissues (N) collected at our hospital. **(D)** Representative images of immunohistochemistry staining in CRC samples with differential protein expression of STK35 collected at our hospital. Scale bar: 100 μm. **(E,F)** Kaplan–Meier plots of **(E)** overall survival rate and **(F)** disease-free survival rate of CRC patients based on differential protein expression of STK35. **(G)** Multivariable analysis performed in the hospital cohort. ** *P* < 0.01, *** *P* < 0.001, compared with N.

To investigate the relationship between STK35 expression and CRC, we collected related mRNA expression data on CRC patients from both online databases and those of our hospital. According to the datasets from TCGA (Figure 1A) and the GSE9348 (Figure 1B), the mRNA expression levels of STK35 in tumor tissues of CRC patients were noticeably (*P* < 0.001 and <0.01, respectively) higher than those in normal tissues. Similarly, based on Q-PCR, we also found that the transcriptional level of STK35 in the 131 tumor tissues from CRC patients in our hospital was significantly (*P* < 0.001) higher than that in the 30 normal tissues (Figure 1C). However, no substantial correlation was found between mRNA expression (median value = 0.9079) and the overall (*P* = 0.095, HR = 1.510, 95% CI: 0.9107–2.504) or disease-free (*P* = 0.1462, HR = 1.476, 95% CI: 0.8780–2.482) survival rates of the CRC patients (Supplementary Figure S1).

Immunohistochemistry staining was used to classified the STK35 protein expression in the CRC patients as high or low, with 78 and 53 cases, respectively (Figure 1D), to explore its correlation with CRC. Kaplan–Meier analysis demonstrated that the protein expression of STK35 was significantly correlated with both the overall (*P* = 0.0197, HR = 1.870, 95% CI: 1.105–3.166; Figure 1E) and disease-free (*P* = 0.0076, HR = 2.078, 95% CI: 1.214–3.558; Figure 1F) survival rates of the CRC patients. Moreover, multivariate analysis revealed that STK35 protein expression was noticeably associated with two clinicopathological parameters in the CRC patients (Table 1), including tumor size (*P* = 0.016) and T classification (*P* = 0.024). Multivariate regression analysis demonstrated that STK35 expression was an independent predictor of CRC aggressiveness, with significant HRs for predicting clinical outcome (Figure 1G). Collectively, STK35 was upregulated and significantly associated with clinicopathologic characteristics, as well as poor prognosis in human CRC.

**TABLE 1:** Correlation between the STK35 protein expression and clinicopathological parameters in patients with colorectal cancer.

Clinicopathological parameter	Protein expression of STK35		*P*-value
	Low	High	
	(*n*=53, 40.4%)	(*n*=78, 59.6%)	
**Gender**			
Male	31 (58.5%)	35 (44.9%)	0.126
Female	22 (41.5%)	43 (55.1%)	
**Age (years)**			
<60	18 (34.0%)	39 (50.0%)	0.069
≥60	35 (66.0%)	39 (50.0%)	
**Tumor size (cm)**			
≤4	31 (58.5%)	29 (37.2%)	0.016*
>4	22 (41.5%)	49 (62.8%)	
**T classification**			
T1	8 (15.1%)	4 (5.1%)	0.024*
T2	15 (28.3%)	11 (14.1%)	
T3	13 (24.5%)	24 (30.8%)	
T4	17 (32.1%)	39 (50.0%)	
**Histology**			
Well	12 (22.6%)	18 (23.1%)	0.326
Moderate	15 (28.3%)	31 (39.7%)	
Poor	26 (49.1%)	29 (37.2%)	

Differences between groups were determined by the Chi-square test, * P<0.05.

### STK35 Knockdown Suppressed CRC Cellular Activities and Tumor Growth

We examined both the mRNA and protein levels of STK35 in a normal human intestinal crypt cell line and various CRC cell lines (Supplementary Figure S2A), following which, we selected SW620 (Supplementary Figure S2B) and HCT116 (Supplementary Figure S2C) cells with the highest expression of STK35 for use in gene knockdown experiments.

**FIGURE 2:**
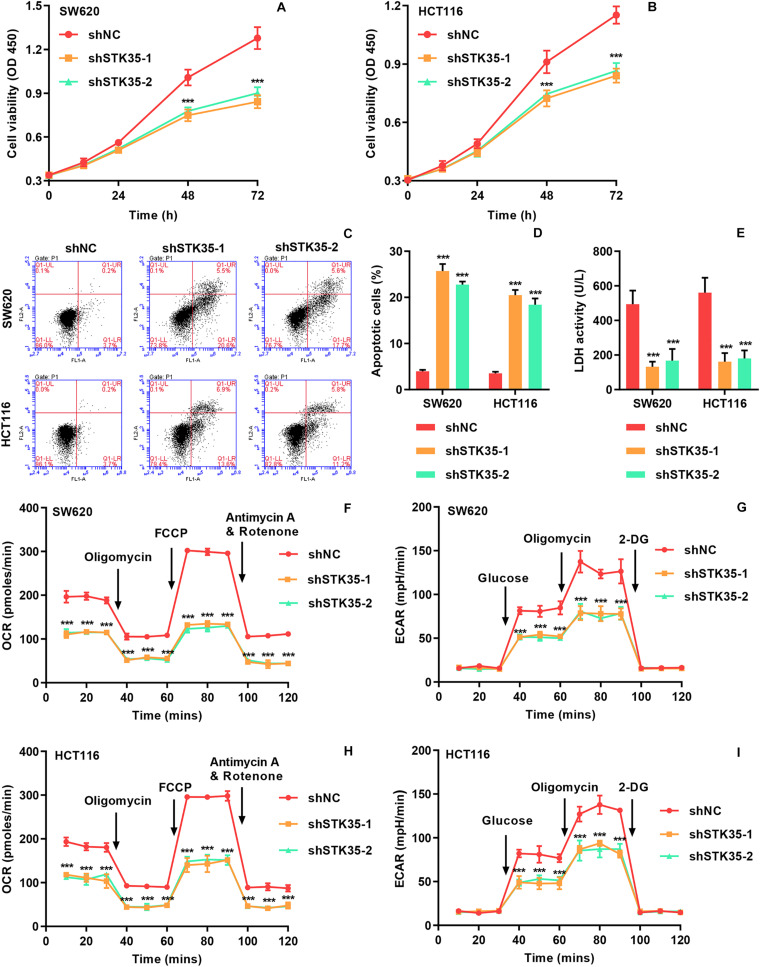
STK35 knockdown inhibits viability and glycolysis but promotes apoptosis in SW620 and HCT116 cells. SW620 and HCT116 cells were transduced with STK35 shRNAs (shSTK35-1 and shSTK35-2) or control scramble shRNA (shNC). **(A,B)** Viability of **(A)** SW620 and **(B)** HCT116 cells assessed by CCK-8. **(C,D)** Assessment and quantification of apoptosis by flow cytometry. **(E)** Cellular LDH levels measured by biochemical analysis. **(F,I)** Energy metabolism of **(F,G)** SW620 and **(H,I)** HCT116 cells reflected by **(F,H)** oxygen consumption rate and **(G,I)** extracellular acidification rate. ***P < 0.001, compared with shNC.

The viability of SW620 (Figure 2A) and HCT116 (Figure 2B) cells with STK35 knockdown was substantially (*P* < 0.001) reduced at 48 and 72 h, compared to that of the control cells. On the contrary, STK35 knockdown significantly (*P* < 0.001) increased apoptosis in both SW620 and HCT116 cells compared to control cells (Figures 2C,D). We also observed that STK35 knockdown in both SW620 and HCT116 cells significantly (*P* < 0.001) decreased their LDH activities compared to control cells (Figure 2E). In addition, the OCR, indicating mitochondrial respiration, and the ECAR, reflecting overall glycolytic flux, were also diminished by STK35 knockdown in both CRC cell lines compared to the control cells (Figures 2F–I).

**FIGURE 3:**
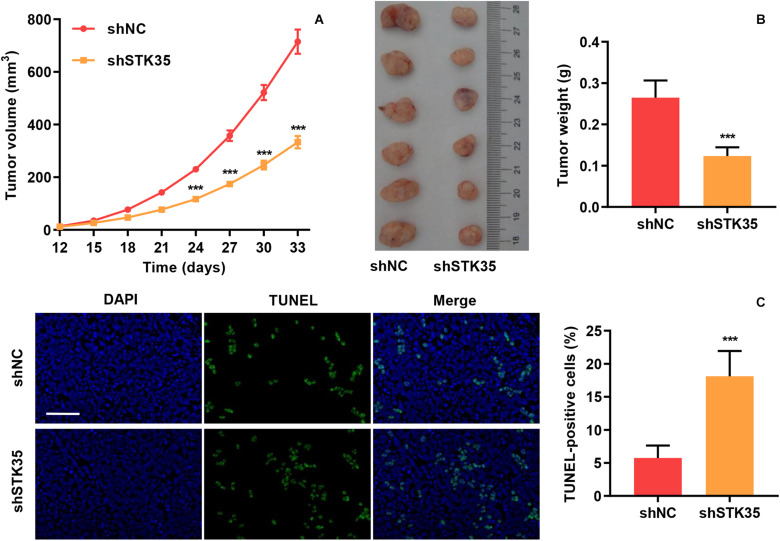
STK35 knockdown constrains tumor growth *in vivo*. SW620 cells stably expressing STK35 shRNAs (shSTK35) or control scramble shRNA (shNC) were subcutaneously injected into male nude mice (*n* = 6 per group). **(A)** Tumor volumes measured every third day from day 12 to 33. **(B)** Mouse tumor characteristics, including morphology and weight, recorded at day 33. **(C)** Visualization of terminal deoxynucleotidyl transferase dUTP nick end labeling (TUNEL) labeled xenograft mouse tumors and quantification of TUNEL positive cells. Scale bar: 100 μm. ***P < 0.001, compared with shNC.

The effect of STK35 on tumor growth was evaluated by subcutaneously injecting nude mice with STK35 knockdown-SW620 cells. We found that in comparison with the control group, the tumor volumes in the SW620 cells with STK35 knockdown-injected mice were notably (*P* < 0.001) reduced from day 24 to 33 (Figure 3A). Similarly, STK35 knockdown notably (*P* < 0.001) reduced the tumor weight at day 33 in relation to the control group (Figure 3B). In contrast, TUNEL analysis demonstrated that STK35 knockdown could significantly (*P* < 0.001) stimulate apoptosis in xenograft mouse tumors (Figure 3C).

### STK35 Regulated Apoptosis, Glycolysis, and AKT Signaling in CRC Cells

**FIGURE 4:**
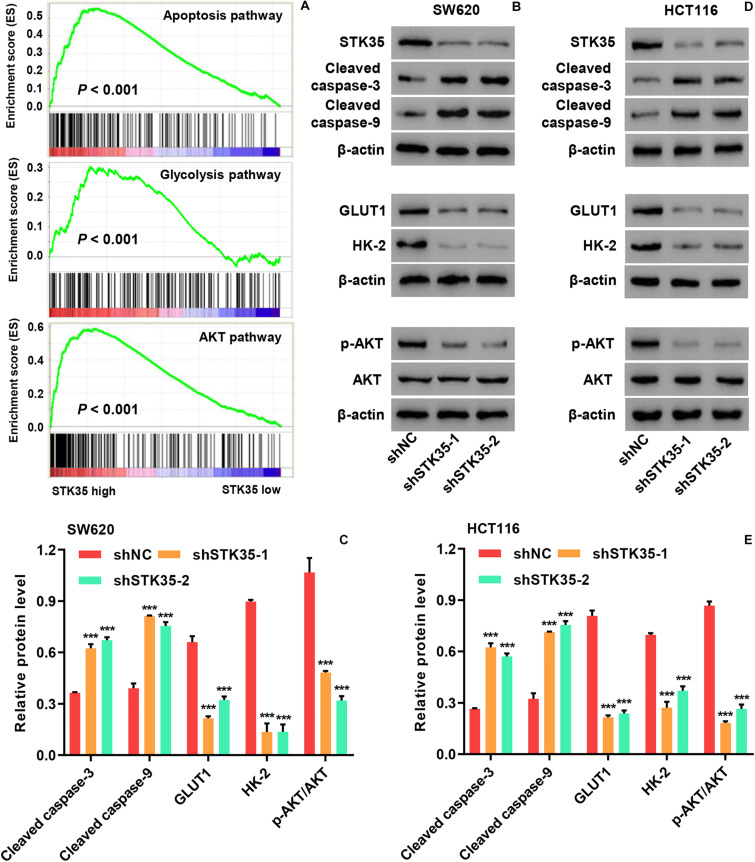
STK35 regulates apoptosis, glycolysis, and AKT signaling in colorectal cancer (CRC). **(A)** Correlation of STK35 high vs. low expression in CRC tissues with genes associated with apoptosis, glycolysis, and AKT pathways, as indicated by gene set enrichment analysis. **(B–E)** Cellular protein levels of STK35, cleaved caspase-3, cleaved caspase-9, GLUT1, HK-2, p-AKT, and AKT in **(B,C)** SW620 and **(D,E)** HCT116 cells transfected with STK35 shRNAs (shSTK35-1 and shSTK35-2) or control scramble shRNA (shNC), assessed by Western blot, with β-actin as the loading control. ***P < 0.001, compared with shNC.

Gene set enrichment analysis demonstrated that the expression of STK35 was significantly (*P* < 0.001) correlated with cancer cell apoptosis, glycolysis, and AKT pathways (Figure 4A). Moreover, Western blot demonstrated that STK35 knockdown in SW620 (Figures 4B,C) and HCT116 (Figures 4D,E) cells led to increased expression of apoptosis-related proteins, such as cleaved caspase-3 and -9. On the contrary, STK35 knockdown in SW620 (Figures 4B,C) and HCT116 (Figures 4D,E) cells led to a reduction in the expression of cellular glycolytic-related proteins, including hexokinase 2 (HK-2) and glucose transporter 1 (GLUT1), as well p-AKT in the AKT signaling pathway. The protein levels of AKT were not altered by STK35 knockdown in either of these cell lines (Figures 4B–E).

### STK35 Overexpression Stimulates CRC Cellular Activities via the AKT Pathway

Based on the protein and mRNA levels of STK35 in the normal human intestinal crypt cell line and various CRC cell lines (Supplementary Figure S2A), we selected SW480 cells with the lowest expression of STK35 for gene overexpression experiments (Supplementary Figure S2D).

**FIGURE 5:**
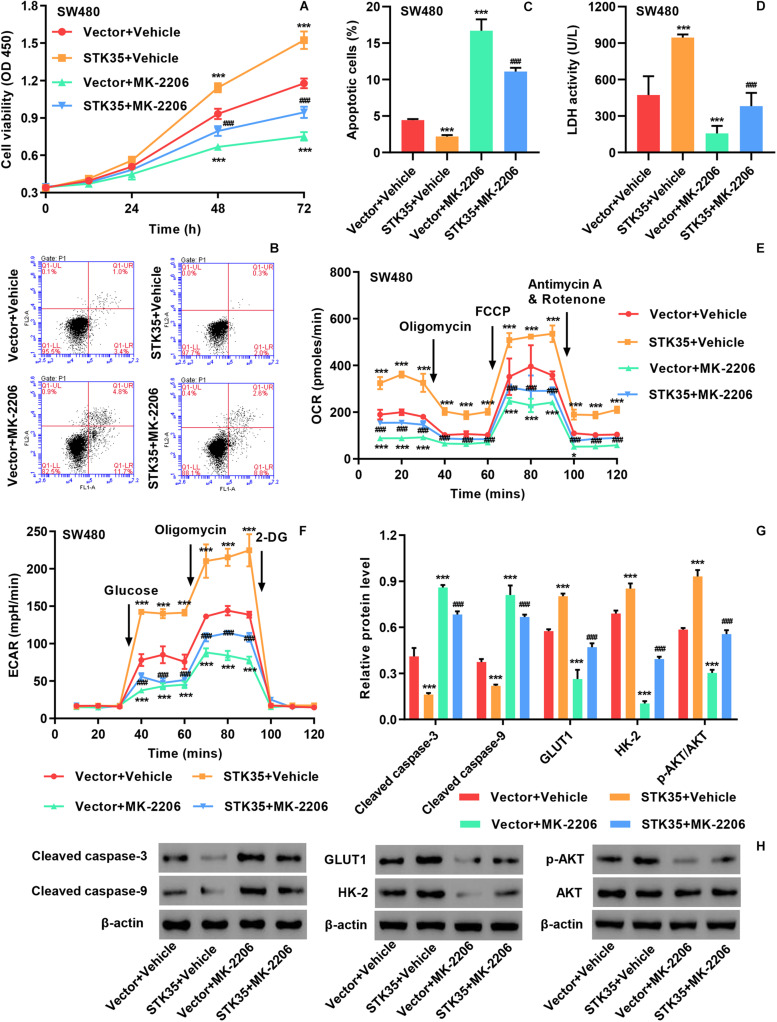
STK35 overexpression promotes viability and glycolysis but inhibits apoptosis in SW480 cells through the AKT signaling pathway. SW480 cells were transduced with STK35 overexpressing lentivirus (STK35) or blank lentivirus (Vector) in the presence of 10 μM AKT inhibitor MK-2206 or control (Vehicle). **(A)** Cell viability assessed by CCK-8. **(B,C)** Cell apoptosis assessed and quantified by flow cytometry. **(D)** Cellular LDH levels measured by biochemical analysis. **(E,F)** Cellular energy metabolism reflected by **(E)** the oxygen consumption rate and **(F)** the extracellular acidification rate. **(G,H)** Cellular protein levels of cleaved caspase-3, cleaved caspase-9, GLUT1, HK-2, p-AKT, and AKT assessed by Western blot, with β-actin as the loading control. *P < 0.05, ***P < 0.001, compared with Vector + Vehicle, ^###^*P* < 0.001, compared with STK35 + Vehicle.

The viability of SW480 cells with STK35 overexpression was significantly (*P* < 0.001) increased at 48 and 72 h compared to that of control cells (Supplementary Figure S3A and Figure 5A). On the contrary, STK35 overexpression substantially (*P* < 0.001) suppressed the apoptosis of SW480 cells compared to the control cells (Supplementary Figure S3B and Figures 5B,C). We also observed that STK35 overexpression in SW480 cells significantly (*P* < 0.001) stimulated their LDH activity in relation to the control cells (Figure 5D). Both the OCR (Figure 5E) and ECAR (Figure 5F) were raised in SW480 cells by STK35 overexpression compared to those in the control cells.

We conducted Western blot to further explore the effects of STK35 overexpression on CRC cellular expression of apoptosis-, glycolysis-, and AKT signaling-related proteins. We found that STK35 overexpression in SW480 cells led to downregulation of cellular cleaved caspase-3 and caspase-9 protein levels, but upregulation of GLUT1 and HK-2 proteins (Figures 5G,H). STK35 overexpression also upregulated the cellular levels of p-AKT protein in SW480 cells in comparison with the control cells, whereas the levels of AKT protein were unchanged (Supplementary Figure S3C and Figures 5G,H).

Through treating the cells with the AKT signaling inhibitor MK-2206, we further demonstrated the molecular mechanism by which STK35 participated in modulating CRC cellular activities. MK-2206 treatment of SW480 cells could significantly (*P* < 0.001) suppress the p-AKT protein level (Figures 5G,H), lower viability at 48 and 72 h (Figure 5A), promote apoptosis (Figure 5B), upregulate apoptosis-related proteins (Figure 5F), reduce LDH activity (Figure 5C), lower the OCR (Figure 5D) and ECAR (Figure 5E), and downregulate glycolytic-related protein levels (Figure 5F) compared to those in the control cells. However, the overexpression of STK35 in MK-2206-treated SW480 cells partially rescued all the observed cellular alterations introduced by the AKT signaling inhibitor at significant levels (*P* < 0.001; Figure 5).

### STK35 Restricted 5-FU Chemosensitivity of CRC

**FIGURE 6:**
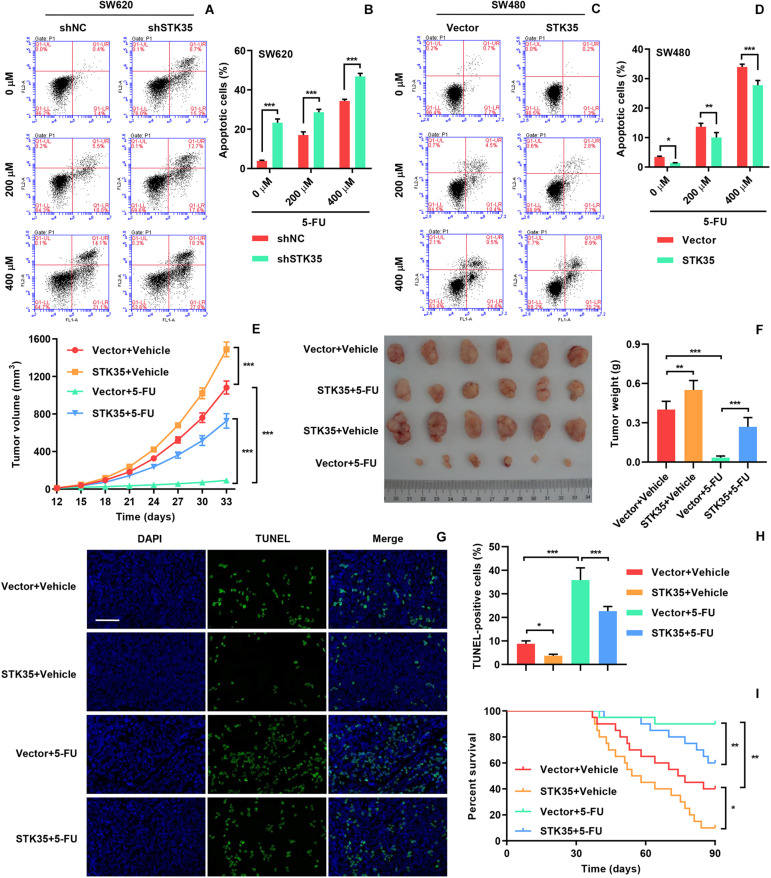
STK35 expression influences chemosensitivity to 5-FU. **(A–D)** SW620 cells stably expressing STK35 shRNAs (shSTK35) or control scramble shRNA (shNC), and SW480 cells transduced with STK35-overexpressing lentivirus (STK35) or blank lentivirus (Vector) were treated with 5-FU (0, 200, and 400 μM) or control (Vehicle) for 48 h. **(A,B)** SW620 and **(C,D)** SW480 apoptosis assessed and quantified by flow cytometry. **(E–I)** SW480 cells transfected with STK35-overexpressing lentivirus (STK35) or blank lentivirus (Vector) following 5-FU chemotherapy were subcutaneously injected into male nude mice (*n* = 6 per group). **(E)** Tumor volumes measured every third day, from days 12 to 33. **(F)** Mouse tumor characteristics, including morphology and weight, recorded at day 33. **(G)** Visualization of terminal deoxynucleotidyl transferase dUTP nick end labeling (TUNEL) labeled xenograft mouse tumors. **(H)** Quantification of TUNEL positive cells. **(I)** Mice survival probability. *P < 0.05, **P < 0.01, ***P < 0.001.

In order to investigate the influence of STK35 on the chemosensitivity of CRC cells, we treated the indicated CRC cells with 5-FU. The 5-FU treatment stimulated apoptosis in both SW620 (Figures 6A,B) and SW480 (Figures 6C,D) cells in a dose-dependent manner. However, STK35 knockdown in 5-FU-treated SW620 cells significantly (*P* < 0.001) strengthened the apoptotic–promotive effect of 5-FU (Figures 6A,B), while STK35 overexpression in 5-FU-treated SW480 cells partially counteracted the effect of 5-FU at significant levels (*P* < 0.01, *P* < 0.001; Figures 6C,D). Following the similar pattern, 5-FU treatment decreased the tumor volumes (Figure 6E) and tumor weight (Figure 6F) in mice, and simultaneously raised the apoptosis in xenograft mouse tumors (Figures 6G,H) and the survival rate of mice (Figure 6I). Nevertheless, STK35 overexpression could partially counteract the antitumor effect of 5-FU at significant levels (Figures 6E–I).

### STK35 Inhibited NEDD4L-Mediated Anti-CRC Effects Through Ubiquitination

**FIGURE 7:**
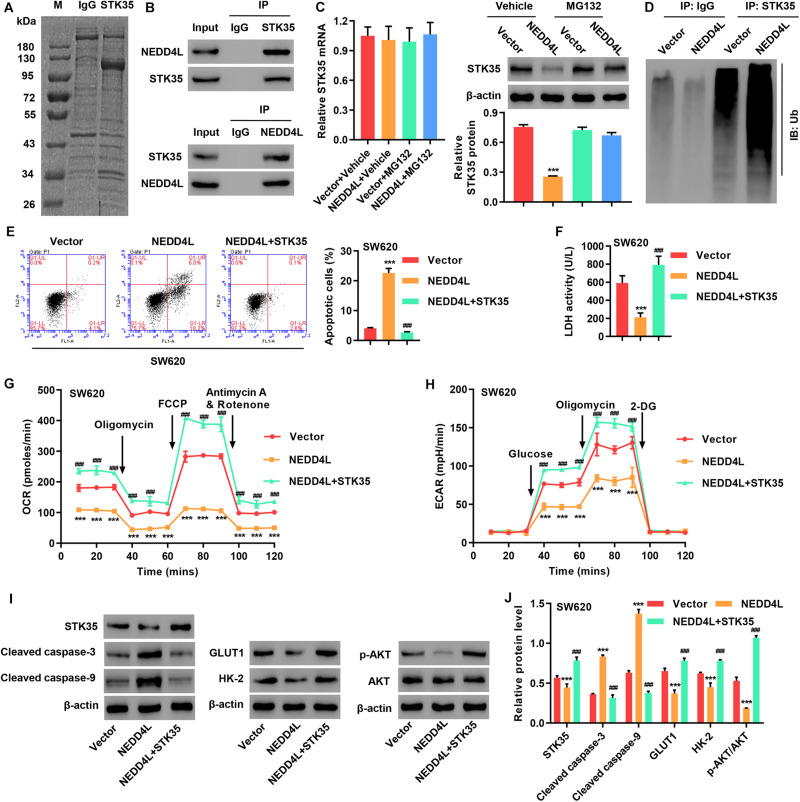
STK35 is ubiquitinated by NEDD4L and inhibits NEDD4L-mediated anti-colorectal cancer function. **(A)** Purification of the STK35 immunocomplex. Proteins were separated by SDS-PAGE and stained with Coomassie Blue. **(B)** Immunoprecipitation was performed with an IgG control, anti-STK35, or anti-NEDD4L antibody, followed by incubation with indicated antibodies. **(C)** Protein and mRNA expression of STK35 in SW620 cells transduced with NEDD4L-overexpressing lentivirus (NEDD4L) or blank lentivirus (Vector) in the presence of 10 μM MG132 or control (Vehicle), measured with western blot and Q-PCR. β-Actin as the loading control. **(D)** NEDD4L-overexpressed SW620 cells immune-precipitated with STK35 or IgG antibody for evaluating ubiquitination. **(E–I)** SW620 cells were transduced with STK35-overexpressing lentivirus (STK35) after NEDD4L-overexpressing lentivirus (NEDD4L) or blank lentivirus (Vector) transfection. **(E)** Cell apoptosis assessed by flow cytometry and quantification accordingly. **(F)** Cellular LDH levels measured by biochemical analysis. **(G,H)** Cellular energy metabolism reflected by **(G)** oxygen consumption rate and **(H)** extracellular acidification rate. **(I,J)** Cellular protein levels of STK35, cleaved caspase-3, cleaved caspase-9, GLUT1, HK-2, p-AKT, and AKT, measured with western blot. β-Actin as the loading control. *** *P* < 0.001, compared with Vector. ^###^ *P* < 0.001, compared with NEDD4L.

To investigate STK35 regulation in CRC, we identified candidate proteins associated with STK35 by Co-immunoprecipitation (Co-IP) assay and proteomics analysis. Differentially expressed bands were excised (Figure 7A) and identified by LC/MS. Among the proteins with ≥3 peptides identified which may be associated with STK35, NEDD4L, which was previously reported to inhibit CRC, was selected for further investigation. We confirmed the association between STK35 and NEDD4L proteins by Co-IP (Figure 7B). Meanwhile, based on the protein and mRNA levels of NEDD4L in the normal human intestinal crypt cell line and various CRC cell lines (Supplementary Figure S2E), we selected SW620 cells with the lowest expression of NEDD4L for overexpression experiments (Supplementary Figure S2F).

To examine the potential STK35 ubiquitination carried out by NEDD4L, we treated the cells with a proteasome inhibitor MG132. The protein expression of STK35 in NEDD4L-overexpressing SW620 cells was downregulated with no significant effect on the STK35 mRNA level; however, treatment with MG132 could restore its expression back to the normal level in the control cells (Figure 7C), suggesting that NEDD4L regulates STK35 levels in a proteasome-dependent manner. Moreover, NEDD4L overexpression in SW620 cells strengthened the ubiquitination of STK35 compared to that of the control cells (Figure 7D).

Most importantly, NEDD4L overexpression in SW620 cells increased apoptosis (*P* < 0.001; Figure 7E), reduced LDH activity (*P* < 0.001; Figure 7F), lowered the OCR (Figure 7G) and ECAR (Figure 7H), upregulated cleaved caspase-3 and -9 cellular protein levels (Figures 7I,J), downregulated GLUT1 and HK-2 protein levels (Figures 7I,J), and suppressed the p-AKT protein level (Figures 7I,J) in relation to those in the control cells. However, further overexpression of STK35 in NEDD4L-overexpressing SW480 cells significantly (*P* < 0.001) counteracted these observed cellular alterations introduced by NEDD4L overexpression (Figures 7E–J).

### STK35 Is Clinically Correlated With NEDD4L and p-AKT in CRC Patients

**FIGURE 8:**
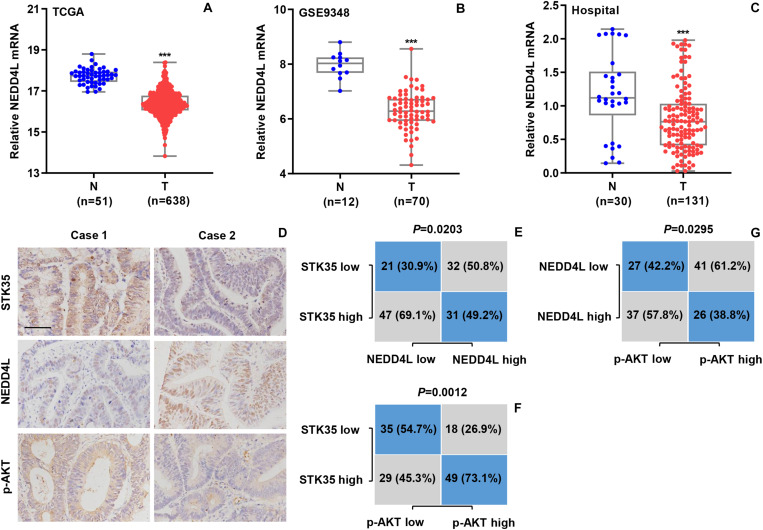
STK35 expression is clinically relevant to NEDD4L and p-AKT in colorectal cancer (CRC). **(A)** NEDD4L expressions in 638 CRC tissues (T) and 51 normal tissues (N) acquired from TCGA RNA-seq datasets. **(B)** NEDD4L expressions in 70 CRC tissues (T) and 12 normal tissues (N) acquired from GSE9348 database. **(C)** NEDD4L expressions assessed by quantitative RT-PCR in 131 CRC tissues (T) and 30 normal tissues (N) collected at our hospital. **(D)** Representative images of immunohistochemistry staining in CRC samples with differential expressions of STK35, NEDD4L, and p-AKT collected at our hospital. Scale bar: 100 μm. **(E–G)** Statistical analyses of CRC tissues for correlation between expressions of **(E)** STK35 and NEDD4L, **(F)** STK35 and p-AKT, and **(G)** NEDD4L and p-AKT. ***P < 0.001, compared with N.

We further explored the relationship between NEDD4L expression and CRC based the mRNA transcriptional data of CRC patients from both online databases and those of our hospital. According to the datasets from TCGA (Figure 8A) and the GSE9348 (Figure 8B), the transcript levels of NEDD4L in tumor tissues from CRC patients were significantly (*P* < 0.001) lower than those in normal tissues. Similarly, based on Q-PCR, we also detected that the transcript level of NEDD4L in the 131 tumor tissues from CRC patients in our hospital was substantially (*P* < 0.001) lower than that in the 30 normal tissues (Figure 8C). Through IHC staining, we classified the NEDD4L and p-AKT protein expressions in the CRC patients as high or low with 63 and 68, and 67 and 64 cases, respectively (Figure 8D). Furthermore, we also revealed significant correlations between STK35 and NEDD4L protein expression (*P* = 0.0203; Figure 8E), STK35 and p-AKT protein expression (*P* = 0.0012; Figure 8F), and NEDD4L and p-AKT protein expression (*P* = 0.0295; Figure 8G).

## Discussion

The expression of human STKs have been reported to be frequently modulated in a variety of human cancers, and particularly, the *STK35* gene is noticeably altered in human CRC (Capra et al., 2006; Goyal et al., 2009; Lu et al., 2017). For the current study, we targeted STK35 based on its regulatory roles in the expression of CDKN2A, with consequent effects on the cell cycle (G1–S phase transition) and programmed cell death (Goyal et al., 2011). Relying on the analyses of patient data collected from both online databases and those of our hospital, we detected a notably higher expression of STK35 in the tumor tissues from CRC patients at both the protein and mRNA levels. Through bioinformatics analysis, the survival probability in patients following CRC diagnosis and treatments was found to be negatively correlated with the protein expression levels of STK35, demonstrating that STK35 could be an indicator of CRC tumor recurrence. This finding is supported by previous reports, which suggested parallel correlations between human STKs and the pathology and prognosis of various types of human cancers, including CRC (Guo et al., 2017; Lu et al., 2017; Sun et al., 2019). Furthermore, multivariate analysis demonstrated a significant association between STK35 protein expression and several CRC clinical parameters, all of which were independently correlated with CRC status in the patients. Together, these results indicate the significance and contribution of STK35 in the clinical management of CRC, for which it could be developed as an efficient prognostic marker.

Previous studies have reported that STK35 and its homolog CLP36 Interacting Kinase 1 Like (Goyal et al., 2009) are capable of regulating a variety of cellular activities in human cancers, in which they have been shown to promote cellular proliferation, migration, and invasion (Yasuda et al., 2012), stimulate the metabolic processes of cancer cells (Zhang et al., 2017), and suppress tumor cell apoptosis (Wu et al., 2018). In the current study, the knockdown of STK35 in CRC cells elevated their apoptotic rate and reduced their proliferation, tumor growth, and energy metabolism, including both mitochondrial respiration and glycolytic flux. In addition, overexpression of STK35 in CRC cells could reduce programmed cell death and increase the tumor-related activities mentioned above. The expression of STK35 was also found to be correlated with cellular apoptosis and glycolysis pathways, which was further confirmed by the elevated apoptotic protein levels and suppressed glycolytic protein levels in CRC cells caused by STK35 knockdown. Hence, these findings jointly demonstrate that STK35 is an important factor in accelerating oncogenesis and the progression of CRC.

Fluorouracil based or adjuvant chemotherapies are well known for their efficacy in the treatment of cancer, and they have been shown to improve survival probability in patients with cancer especially CRC, as a result of regulating the folate metabolic pathway and inhibiting both DNA and RNA synthesis (Pardini et al., 2011; Hamaya et al., 2015). However, the presence of tumor stem cells possessing chemoresistance, which accounts for approximately 0.05–0.1% of the total tumor cell mass, and harbor unlimited self-renewal competency, is the main cause of failure of cancer chemotherapy (Zeuner et al., 2014). As a matter of fact, a great number of human kinases have been identified as coexistent putative markers of tumor stem cells, which contribute to minimizing 5-FU chemosensitivity in human cancers (Li et al., 2019; Hou et al., 2020). In the current study, we observed the anti-CRC functions of 5-FU in a dose-dependent manner as expected, whereas the overexpression of STK35 in CRC cells partially reversed these effects, such as the promotion of apoptosis, tumor growth inhibition, and survival improvement in an *in vivo* mouse model. Additionally, previous studies have illustrated that reprogrammed glycolysis, as a recognized hallmark for malignancy which generates plentiful intermediate functional products, also contributes to cancer resistance toward therapeutic drugs (Qian et al., 2017; Ma and Zong, 2020), which is in accordance with our findings in the present study. All these results suggest that STK35 is capable of influencing CRC chemoresistance toward 5-FU, partially attributed to its role in the induction of the glycolytic process in cancerous cells, while, to a further extent, targeting STK35 downregulation in CRC patients might improve the efficacy of chemotherapy.

The phosphorylation of AKT, forming p-AKT, is a crucial step for activating the PI3K/AKT signaling pathway in oncogenesis (Chang et al., 2003). Using GSEA, we have identified the positive correlation between STK35 expression and the AKT pathway. The AKT pathway is also involved in modulating chemoresistance of human cancer cells (Deng et al., 2019), which, together with the findings on STK35-CRC resistance toward 5-FU, indicates close relationships among STK35, the AKT signaling pathway, and CRC chemoresistance. Moreover, the knockdown and overexpression of STK35 in CRC cells induced the downregulation and upregulation of cellular p-AKT, respectively, further confirming the connection between STK35 and AKT phosphorylation. Indeed, complete activation of the core molecule AKT, also an STK (Osaki et al., 2004), is fulfilled by the phosphorylation of its threonine Thr308 and serine Ser473 sites (Liao and Hung, 2010). According to previous studies, the phosphorylation of AKT at either site could be effectively modulated by multiple kinases and phosphatases (Zhang et al., 2006; Kanan et al., 2010). In this study, we observed that STK35 overexpression could partly counteract the anti-CRC functions induced by the AKT inhibitor MK-2206, further illustrating that STK35 may promote the activities of CRC cells via its regulatory effect on the AKT pathway. However, further studies are necessary to discover the exact mechanism by which STK35 regulates AKT/p-AKT, either directly or indirectly.

Relying on the results of the proteomics-based analysis, we identified that NEDD4L interacts with STK35, which was further validated by Co-IP. NEDD4L functions as an E3 ubiquitin ligase and plays essential roles in malignancy and tumorigenesis (Rotin and Kumar, 2009). Moreover, NEDD4L was previously reported to inhibit CRC through suppression of the canonical WNT signaling pathway (Tanksley et al., 2013), and its expression could be targeted as either a prognostic or therapeutic biomarker (Ye et al., 2014). We also detected a high transcriptional level of NEDD4L in CRC tumor tissues and found that its overexpression in CRC cells could promote cellular apoptosis, suppress the glycolytic process, influence the expression of key proteins, and modulate the p-AKT protein level. Additionally, NEDD4L is known to catalyze the polyubiquitination of multiple critical kinases, particularly STKs, to regulate the cell cycle and prevent carcinogenesis (Escobedo et al., 2014). In the current study, NEDD4L overexpression in CRC cells downregulated the cellular level of STK35, which was reversed by the proteasome inhibitor MG132, indicating that NEDD4L may also mediate the ubiquitination of STK35. Indeed, the overexpression of NEDD4L in CRC cells intensified STK35 ubiquitination, which unerringly confirmed our speculation. NEDD4L and STK35 have opposite regulatory functions on the PI3K/AKT pathway (Rotin and Kumar, 2009; Ye et al., 2014), the former of which ubiquitinates PI3K and maintains the balanced state of the pathway (Wang et al., 2016), while the latter activates the pathway. Therefore, STK35 overexpression was capable of completely offsetting the observed anti-CRC effects exhibited by NEDD4L overexpression, and it simultaneously recued the cellular p-AKT back to an even higher level. We have also successfully uncovered substantial correlations among the protein expression of STK35, NEDD4L, and p-AKT. These discoveries highlight the NEDD4L-mediated ubiquitination and degradation of STK35, by which STK35 counteracts NEDD4L-induced anticancer functions partially through regulating the AKT signaling pathway.

To the best of our knowledge, the current study is the first to demonstrate the roles of STK35 in CRC and their related molecular mechanisms. In summary, we reveal higher overall expression of STK35 in CRC tissues, its substantial correlation with CRC prognosis, and its suppressive effects on CRC cell apoptosis. In addition, we demonstrate that STK35 promotes CRC cellular viability, energy metabolism, tumor growth, and chemoresistance. We speculate that STK35 could modulate these CRC activities partially through regulating the AKT signaling pathway. Moreover, the E3 ubiquitin ligase NEDD4L was revealed to associate and ubiquitinate STK35, and its anti-CRC functions were found to be counteracted by STK35. Based on these findings, this study establishes the foundation for locating therapeutic targets in CRC therapy and developing CRC prognostic biomarkers in clinical management.

## Data Availability Statement

All datasets presented in this study are included in the article/Supplementary Material.

## Ethics Statement

The studies involving human participants were reviewed and approved by The Affiliated Changzhou No. 2 People’s Hospital of Nanjing Medical University. The patients/participants provided their written informed consent to participate in this study. The animal study was reviewed and approved by The Affiliated Changzhou No. 2 People’s Hospital of Nanjing Medical University.

## Author Contributions

HY and JZ conceived and designed the work. JZ, GW, and HL performed the research and collected and analyzed the data. GW and YZ collected the human tissue samples. HL, YZ, and JQ provided the technical assistance. HY and JQ wrote the manuscript. All authors read and approved the final manuscript.

## Conflict of Interest

The authors declare that the research was conducted in the absence of any commercial or financial relationships that could be construed as a potential conflict of interest.

## References

[B1] Arnold M. Sierra M. S. Laversanne M. Soerjomataram I. Jemal A. Bray F. (2017). Global patterns and trends in colorectal cancer incidence and mortality. *Gut* 66 683–691. 10.1136/gutjnl-2015-310912 26818619

[B2] Brenner H. Kloor M. Pox C. P. (2014). Colorectal cancer. *Lancet* 383 1490–1502. 24225001 10.1016/S0140-6736(13)61649-9

[B3] Brown R. E. Short S. P. Williams C. S. (2018). Colorectal cancer and metabolism. *Curr. Colorectal Cancer Rep.* 14 226–241. 10.1007/s11888-018-0420-y 31406492 PMC6690608

[B4] Capra M. Nuciforo P. G. Confalonieri S. Quarto M. Bianchi M. Nebuloni M. (2006). Frequent alterations in the expression of serine/threonine kinases in human cancers. *Cancer Res.* 66 8147–8154. 10.1158/0008-5472.can-05-3489 16912193

[B5] Chang F. Lee J. T. Navolanic P. M. Steelman L. S. Shelton J. G. Blalock W. L. (2003). Involvement of PI3K/Akt pathway in cell cycle progression, apoptosis, and neoplastic transformation: a target for cancer chemotherapy. *Leukemia* 17 590–603. 10.1038/sj.leu.2402824 12646949

[B6] Cheung T. H. Rando T. A. (2013). Molecular regulation of stem cell quiescence. *Nat. Rev. Mol. Cell Biol.* 14 329–340. 10.1038/nrm3591 23698583 PMC3808888

[B7] Cronin K. A. Lake A. J. Scott S. Sherman R. L. Noone A. M. Howlader N. (2018). Annual report to the nation on the status of cancer, part I: national cancer statistics. *Cancer* 124 2785–2800. 10.1002/cncr.31551 29786848 PMC6033186

[B8] Danielsen S. A. Eide P. W. Nesbakken A. Guren T. Leithe E. Lothe R. A. (2015). Portrait of the PI3K/AKT pathway in colorectal cancer. *Biochim. Biophys. Acta* 1855 104–121. 10.1016/j.bbcan.2014.09.008 25450577

[B9] Deng J. Bai X. Feng X. Ni J. Beretov J. Graham P. (2019). Inhibition of PI3K/Akt/mTOR signaling pathway alleviates ovarian cancer chemoresistance through reversing epithelial-mesenchymal transition and decreasing cancer stem cell marker expression. *BMC Cancer* 19:618. 10.1186/s12885-019-5824-9 31234823 PMC6591840

[B10] Escobedo A. Gomes T. Aragon E. Martin-Malpartida P. Ruiz L. Macias M. J. (2014). Structural basis of the activation and degradation mechanisms of the E3 ubiquitin ligase Nedd4L. *Structure* 22 1446–1457. 10.1016/j.str.2014.08.016 25295397

[B11] Fearon E. R. Vogelstein B. (1990). A genetic model for colorectal tumorigenesis. *Cell* 61 759–767. 10.1016/0092-8674(90)90186-i 2188735

[B12] Freeman S. M. Whartenby K. A. (2004). The role of the mitogen-activated protein kinase cellular signaling pathway in tumor cell survival and apoptosis. *Drug News Perspect.* 17 237–242. 10.1358/dnp.2004.17.4.829050 15334172

[B13] Goyal P. Behring A. Kumar A. Siess W. (2009). Identifying and characterizing a novel protein kinase STK35L1 and deciphering its orthologs and close-homologs in vertebrates. *PLoS One* 4:e6981. 10.1371/journal.pone.0006981 19756140 PMC2737284

[B14] Goyal P. Behring A. Kumar A. Siess W. (2011). STK35L1 associates with nuclear actin and regulates cell cycle and migration of endothelial cells. *PLoS One* 6:e16249. 10.1371/journal.pone.0016249 21283756 PMC3024402

[B15] Guo Y. Liang F. Zhao F. Zhao J. (2020). Resibufogenin suppresses tumor growth and Warburg effect through regulating miR-143-3p/HK2 axis in breast cancer. *Mol. Cell Biochem.* 466 103–115. 10.1007/s11010-020-03692-z 32006291

[B16] Guo Z. Peng G. Li E. Xi S. Zhang Y. Li Y. (2017). MAP kinase-interacting serine/threonine kinase 2 promotes proliferation, metastasis, and predicts poor prognosis in non-small cell lung cancer. *Sci. Rep.* 7:10612. 10.1038/s41598-017-10397-9 PMC5587555 28878291

[B17] Hamaya Y. Guarinos C. Tseng-Rogenski S. S. Iwaizumi M. Das R. Jover R. (2015). Efficacy of adjuvant 5-fluorouracil therapy for patients with EMAST-positive stage II/III colorectal cancer. *PLoS One* 10:e0127591 10.1371/journal.pone.00127591 PMC4440728 25996601

[B18] Hou J. Tan Y. Su C. Wang T. Gao Z. Song D. (2020). Inhibition of protein FAK enhances 5-FU chemosensitivity to gastric carcinoma via p53 signaling pathways. *Comput. Struct. Biotechnol. J.* 18 125–136. 10.1016/j.csbj.2019.12.010 31969973 PMC6961071

[B19] Hourani M. Berretta R. Mendes A. Moscato P. (2008). Genetic signatures for a rodent model of Parkinson’s disease using combinatorial optimization methods. *Methods Mol. Biol.* 453 379–392. 10.1007/978-1-60327-429-6_20 18712315

[B20] Hung Y. P. Teragawa C. Kosaisawe N. Gillies T. E. Pargett M. Minguet M. (2017). Akt regulation of glycolysis mediates bioenergetic stability in epithelial cells. *eLife* 6:e27293. 10.7554/eLife.27293 29239720 PMC5730373

[B21] Kanan Y. Matsumoto H. Song H. Sokolov M. Anderson R. E. Rajala R. V. (2010). Serine/threonine kinase akt activation regulates the activity of retinal serine/threonine phosphatases, PHLPP and PHLPPL. *J. Neurochem.* 113 477–488. 10.1111/j.1471-4159.2010.06609.x 20089132 PMC2909469

[B22] La Vecchia S. Sebastian C. (2020). Metabolic pathways regulating colorectal cancer initiation and progression. *Semin. Cell Dev. Biol.* 98 63–70. 10.1016/j.semcdb.2019.05.018 31129171

[B23] Li L. Jones K. Mei H. (2019). Doublecotin-Like Kinase 1 increases chemoresistance of colorectal cancer cells through the anti-apoptosis pathway. *J. Stem Cell Res. Ther.* 9:447. 10.4172/2157-7633.1000447 PMC6671676 31372308

[B24] Liao Y. Hung M. C. (2010). Physiological regulation of Akt activity and stability. *Am. J. Transl. Res.* 2 19–42. 20182580 PMC2826820

[B25] Lu Y. Tang J. Zhang W. Shen C. Xu L. Yang D. (2017). Correlation between STK33 and the pathology and prognosis of lung cancer. *Oncol. Lett.* 14 4800–4804. 10.3892/ol.2017.6766 29085482 PMC5649584

[B26] Ma L. Zong X. (2020). Metabolic symbiosis in chemoresistance: refocusing the role of aerobic glycolysis. *Front. Oncol.* 10:5. 10.3389/fonc.2020.00005 32038983 PMC6992567

[B27] Malinowsky K. Nitsche U. Janssen K. P. Bader F. G. Spath C. Drecoll E. (2014). Activation of the PI3K/AKT pathway correlates with prognosis in stage II colon cancer. *Br. J. Cancer* 110 2081–2089. 10.1038/bjc.2014.100 24619078 PMC3992486

[B28] Manning B. D. Cantley L. C. (2007). AKT/PKB signaling: navigating downstream. *Cell* 129 1261–1274. 10.1016/j.cell.2007.06.009 17604717 PMC2756685

[B29] Manning G. Whyte D. B. Martinez R. Hunter T. Sudarsanam S. (2002). The protein kinase complement of the human genome. *Science* 298 1912–1934. 10.1126/science.1075762 12471243

[B30] Nitulescu G. M. Van De Venter M. Nitulescu G. Ungurianu A. Juzenas P. Peng Q. (2018). The Akt pathway in oncology therapy and beyond (Review). *Int. J. Oncol.* 53 2319–2331. 30334567 10.3892/ijo.2018.4597 PMC6203150

[B31] Osaki M. Oshimura M. Ito H. (2004). PI3K-Akt pathway: its functions and alterations in human cancer. *Apoptosis* 9 667–676. 10.1023/b:appt.0000045801.15585.dd 15505410

[B32] Pardini B. Kumar R. Naccarati A. Novotny J. Prasad R. B. Forsti A. (2011). 5-Fluorouracil-based chemotherapy for colorectal cancer and MTHFR/MTRR genotypes. *Br. J. Clin. Pharmacol.* 72 162–163. 10.1111/j.1365-2125.2010.03892.x 21204909 PMC3141199

[B33] Qian X. Xu W. Xu J. Shi Q. Li J. Weng Y. (2017). Enolase 1 stimulates glycolysis to promote chemoresistance in gastric cancer. *Oncotarget* 8 47691–47708. 10.18632/oncotarget.17868 28548950 PMC5564598

[B34] Rotin D. Kumar S. (2009). Physiological functions of the HECT family of ubiquitin ligases. *Nat. Rev. Mol. Cell Biol.* 10 398–409. 10.1038/nrm2690 19436320

[B35] Siegel R. L. Miller K. D. Fedewa S. A. Ahnen D. J. Meester R. G. S. Barzi A. (2017). Colorectal cancer statistics, 2017. *CA Cancer J. Clin.* 67 177–193. 10.3322/caac.21395 28248415

[B36] Slattery M. L. Mullany L. E. Wolff R. K. Sakoda L. C. Samowitz W. S. Herrick J. S. (2019). The p53-signaling pathway and colorectal cancer: interactions between downstream p53 target genes and miRNAs. *Genomics* 111 762–771. 10.1016/j.ygeno.2018.05.006 29860032 PMC6274615

[B37] Sun E. Liu K. Zhao K. Wang L. (2019). Serine/threonine kinase 32C is overexpressed in bladder cancer and contributes to tumor progression. *Cancer Biol. Ther.* 20 307–320. 10.1080/15384047.2018.1529098 30359551 PMC6370379

[B38] Tanksley J. P. Chen X. Coffey R. J. (2013). NEDD4L is downregulated in colorectal cancer and inhibits canonical WNT signaling. *PLoS One* 8:e81514. 10.1371/journal.pone.0081514 24312311 PMC3842946

[B39] Vallenius T. Makela T. P. (2002). Clik1: a novel kinase targeted to actin stress fibers by the CLP-36 PDZ-LIM protein. *J. Cell Sci.* 115 2067–2073. 11973348 10.1242/jcs.115.10.2067

[B40] Vander Heiden M. G. Cantley L. C. Thompson C. B. (2009). Understanding the Warburg effect: the metabolic requirements of cell proliferation. *Science* 324 1029–1033. 10.1126/science.1160809 19460998 PMC2849637

[B41] Wang Z. Dang T. Liu T. Chen S. Li L. Huang S. (2016). NEDD4L protein catalyzes ubiquitination of PIK3CA protein and regulates PI3K-AKT signaling. *J. Biol. Chem.* 291 17467–17477. 10.1074/jbc.m116.726083 27339899 PMC5016142

[B42] Wu Z. Liu J. Hu S. Zhu Y. Li S. (2018). Serine/Threonine Kinase 35, a Target Gene of STAT3, regulates the proliferation and apoptosis of osteosarcoma cells. *Cell Physiol. Biochem.* 45 808–818. 10.1159/000487172 29414823

[B43] Yasuda Y. Miyamoto Y. Yamashiro T. Asally M. Masui A. Wong C. (2012). Nuclear retention of importin alpha coordinates cell fate through changes in gene expression. *EMBO J.* 31 83–94. 10.1038/emboj.2011.360 21964068 PMC3252573

[B44] Ye X. Wang L. Shang B. Wang Z. Wei W. (2014). NEDD4: a promising target for cancer therapy. *Curr. Cancer Drug Targets* 14 549–556. 10.2174/1568009614666140725092430 25088038 PMC4302323

[B45] Yu M. Grady W. M. (2012). Therapeutic targeting of the phosphatidylinositol 3-kinase signaling pathway: novel targeted therapies and advances in the treatment of colorectal cancer. *Therap. Adv. Gastroenterol.* 5 319–337. 10.1177/1756283x12448456 22973417 PMC3437536

[B46] Zeuner A. Todaro M. Stassi G. De Maria R. (2014). Colorectal cancer stem cells: from the crypt to the clinic. *Cell Stem Cell* 15 692–705. 10.1016/j.stem.2014.11.012 25479747

[B47] Zhang L. Yang H. Zhang W. Liang Z. Huang Q. Xu G. (2017). Clk1-regulated aerobic glycolysis is involved in glioma chemoresistance. *J. Neurochem.* 142 574–588. 10.1111/jnc.14096 28581641

[B48] Zhang Q. Adiseshaiah P. Kalvakolanu D. V. Reddy S. P. (2006). A Phosphatidylinositol 3-kinase-regulated Akt-independent signaling promotes cigarette smoke-induced FRA-1 expression. *J. Biol. Chem.* 281 10174–10181. 10.1074/jbc.m513008200 16490785

[B49] Zheng H. C. (2017). The molecular mechanisms of chemoresistance in cancers. *Oncotarget* 8 59950–59964. 10.18632/oncotarget.19048 28938696 PMC5601792

[B50] Zhu W. Li Z. Xiong L. Yu X. Chen X. Lin Q. (2017). FKBP3 promotes proliferation of non-small cell lung cancer cells through regulating Sp1/HDAC2/p27. *Theranostics* 7 3078–3089. 10.7150/thno.18067 28839465 PMC5566107

