# Supplementary material for: STK35 Is Ubiquitinated by NEDD4L and Promotes Glycolysis and Inhibits Apoptosis Through Regulating the AKT Signaling Pathway, Influencing Chemoresistance of Colorectal Cancer
Source: Front Cell Dev Biol. 2020 Oct 8;8:582695. doi: 10.3389/fcell.2020.582695 (PMC7578231; doi:10.3389/fcell.2020.582695)
Supplement: Supplementary file 1 [file Data_Sheet_1.docx]

**STK35 is ubiquitinated by NEDD4L, and promotes glycolysis, and inhibits apoptosis through regulating the AKT signaling pathway, influencing chemoresistance of colorectal cancer**

Haojun Yang*, Jie Zhu*, Guangyao Wang, Hanyang Liu, Yan Zhou, Jun Qian

Department of Gastrointestinal Center, The Affiliated Changzhou NO.2 People's Hospital of Nanjing Medical University, Changzhou 213003, China

* Contributed equally

Corresponding author

Jun Qian, Department of Gastrointestinal Center, The Affiliated Changzhou No. 2 People's Hospital of Nanjing Medical University, 68th Gehu Road, Wujin district, Changzhou 213003, China. Tel: +86-0519-81087310; E-mail: [drqianjun@sina.com](mailto:drqianjun@sina.com)

**Running title:** STK35 ubiquitinated by NEDD4L regulates the AKT pathway

**
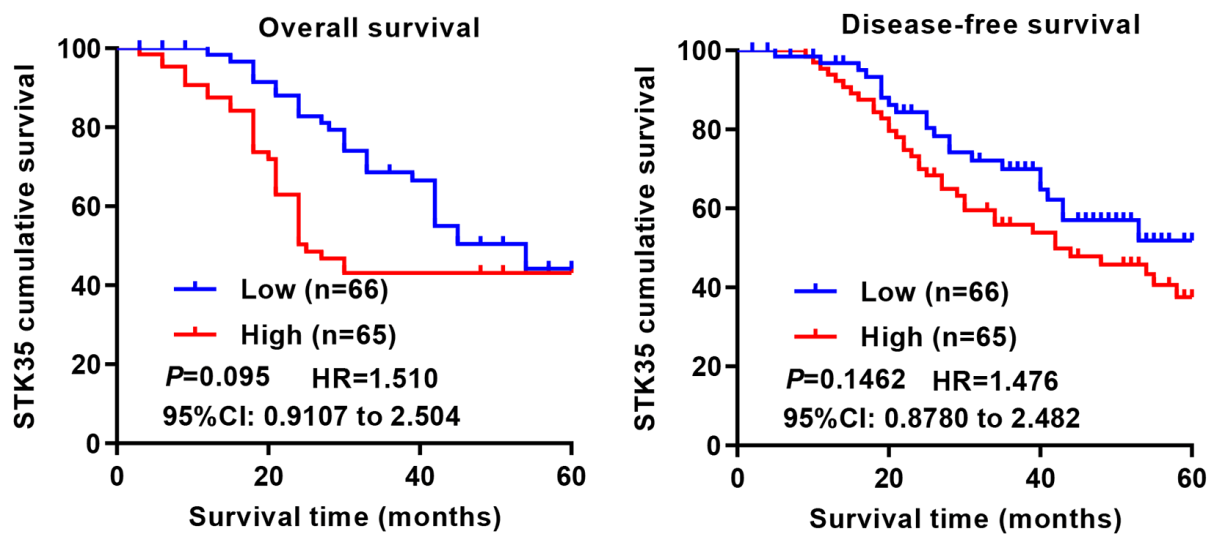
**

**Figure S1. STK35 is not clinically correlated with survival probability of colorectal cancer (CRC) patients.** Kaplan-Meier plots for overall survival rate and disease-free survival rate of CRC patients based on differential mRNA expressions of STK35 according to Figure 1C.


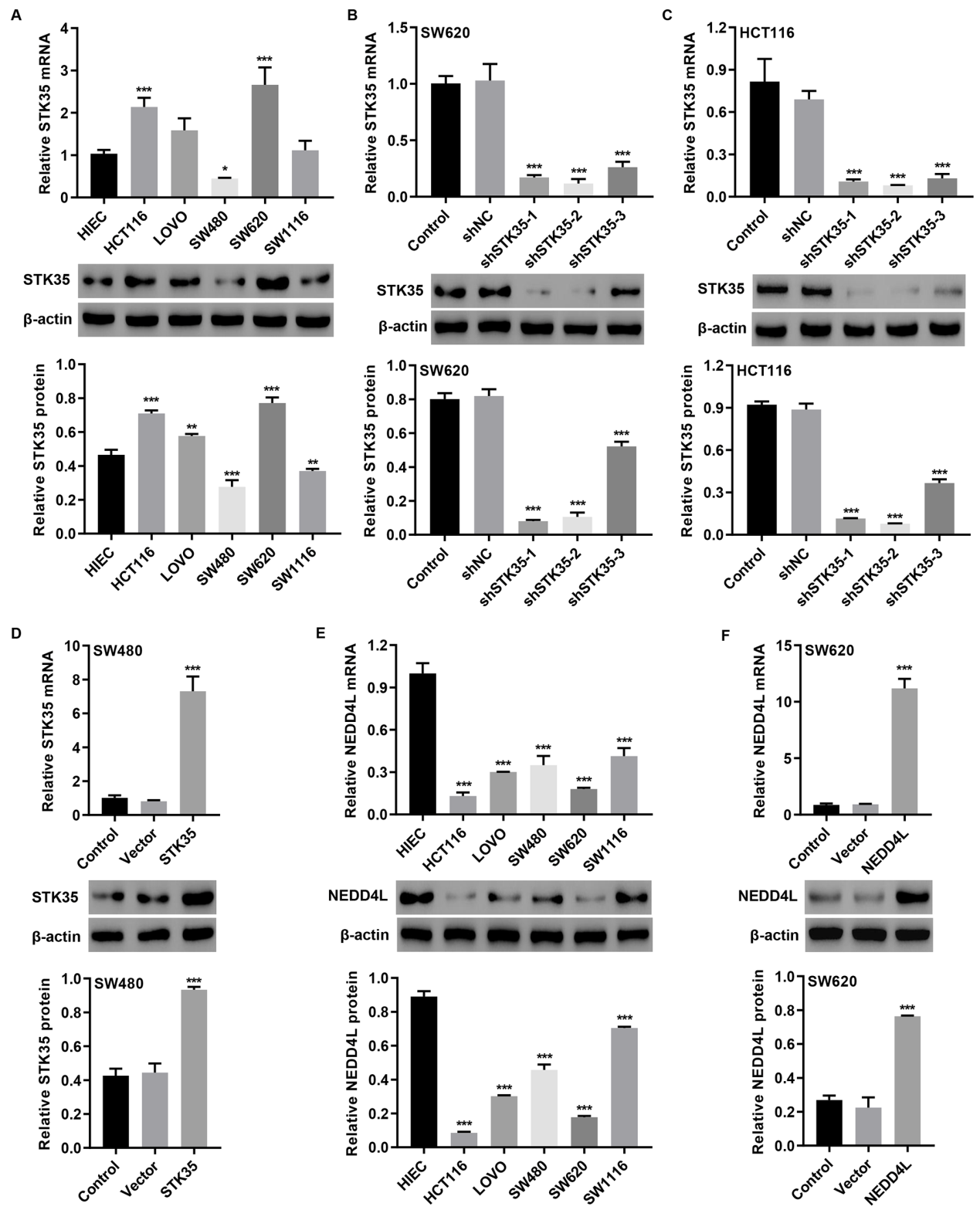


**Figure S2. STK35 and NEDD4L expressions in various cell lines.** (A) The protein and mRNA expression levels of STK35 in colorectal cancer (CRC) cell lines (HCT116, LOVO, SW480, SW620, and SW1116) and normal human intestinal crypt cell line (HIEC). (B) The mRNA and protein levels of STK35 in (B) SW620 and (C) HCT116 cells transduced with STK35 shRNAs (shSTK35-1, shSTK35-2, and shSTK35-3) or control scramble shRNA (shNC). (D) The protein and mRNA expression levels of STK35 in SW480 cells transduced with STK35 overexpressing lentivirus (STK35) or blank lentivirus (Vector). (E) The protein and mRNA expression levels of NEDD4L in CRC cell lines (HCT116, LOVO, SW480, SW620, and SW1116) and normal human intestinal crypt cell line (HIEC). (F) The protein and mRNA expression levels of NEDD4L in SW620 cells transduced with NEDD4L overexpressing lentivirus (NEDD4L) or blank lentivirus (Vector). * *P*<0.05, ** *P*<0.01, *** *P*<0.001, compared with HIEC or control.


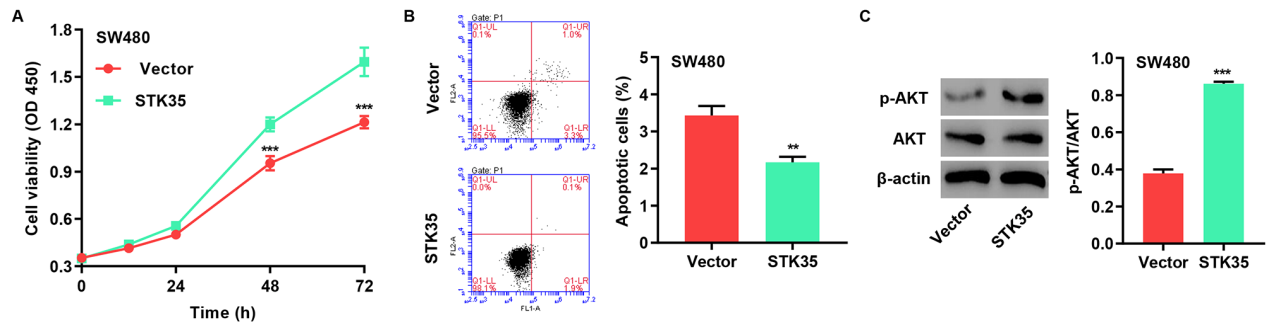


**Figure S3. STK35 overexpression promotes viability, inhibits apoptosis in SW480 cells, and regulates AKT signaling pathway.** SW480 cells were transduced with STK35 overexpressing lentivirus (STK35) or blank lentivirus (Vector). (A) Cell viability assessed by CCK-8. (B) Cell apoptosis assessed by flow cytometry and quantification accordingly. (C) Cellular protein levels of p-AKT and AKT, measured with western blot. β-actin as the loading control. ** *P*<0.01, *** *P*<0.001, compared with Vector.
